# Ten-Year Results of FAST: A Randomized Controlled Trial of 5-Fraction Whole-Breast Radiotherapy for Early Breast Cancer

**DOI:** 10.1200/JCO.19.02750

**Published:** 2020-07-14

**Authors:** Adrian Murray Brunt, Joanne S. Haviland, Mark Sydenham, Rajiv K. Agrawal, Hafiz Algurafi, Abdulla Alhasso, Peter Barrett-Lee, Peter Bliss, David Bloomfield, Joanna Bowen, Ellen Donovan, Andy Goodman, Adrian Harnett, Martin Hogg, Sri Kumar, Helen Passant, Mary Quigley, Liz Sherwin, Alan Stewart, Isabel Syndikus, Jean Tremlett, Yat Tsang, Karen Venables, Duncan Wheatley, Judith M. Bliss, John R. Yarnold

**Affiliations:** ^1^ Cancer Centre, University Hospitals of North Midlands NHS Trust and Keele University, Stoke-on-Trent, Staffordshire, United Kingdom; ^2^ Clinical Trials and Statistics Unit, Institute of Cancer Research, Sutton, London, United Kingdom; ^3^ Oncology Centre, Lingen Davies Centre, Royal Shrewsbury Hospital, Shrewsbury, Shropshire, United Kingdom; ^4^ Oncology Department, Southend University Hospital, Southend, Essex, United Kingdom; ^5^ Radiotherapy, Beatson West of Scotland Cancer Centre, Glasgow, Scotland; ^6^ Velindre Cancer Centre, Velindre Hospital, Cardiff, Wales; ^7^ Oncology, Torbay Hospital, Torquay, Devon, United Kingdom; ^8^ Sussex Cancer Centre, Royal Sussex County Hospital, Brighton, Sussex, United Kingdom; ^9^ Oncology Centre, Cheltenham General Hospital, Cheltenham, Gloucestershire, United Kingdom; ^10^ Centre for Vision, Speech, and Signal Processing, University of Surrey, Guildford, Surrey, United Kingdom; ^11^ Exeter Oncology Centre, Royal Devon and Exeter Hospital, Exeter, Devon, United Kingdom; ^12^ Oncology and Haematology Department, Norfolk and Norwich University Hospital, Norwich, Norfolk, United Kingdom; ^13^ The Cancer Centre, Royal Preston Hospital, Preston, Lancashire, United Kingdom; ^14^ Leeds Cancer Centre, St James’s University Hospital, Leeds, Yorkshire, United Kingdom; ^15^ Oncology Department, Queen’s Hospital, Romford, Essex, United Kingdom; ^16^ Department of Oncology and Haematology, Ipswich Hospital, Ipswich, Suffolk, United Kingdom; ^17^ Radiotherapy Department, The Christie Hospital, Manchester, Lancashire, United Kingdom; ^18^ The Clatterbridge Cancer Centre, Clatterbridge Hospital, Bebington, Wirral, Cheshire, United Kingdom; ^19^ RTTQA, Mount Vernon Hospital, Rickmansworth, Middlesex, United Kingdom; ^20^ The Sunrise Centre, Royal Cornwall Hospital, Truro, Cornwall, United Kingdom; ^21^ Institute of Cancer Research and Royal Marsden Hospital NHS Foundation Trust, Sutton, Surrey, United Kingdom

## Abstract

**PURPOSE:**

Previous studies of hypofractionated adjuvant whole-breast radiotherapy for early breast cancer established a 15- or 16-fraction (fr) regimen as standard. The FAST Trial (CRUKE/04/015) evaluated normal tissue effects (NTE) and disease outcomes after 5-fr regimens. Ten-year results are presented.

**METHODS:**

Women ≥ 50 years of age with low-risk invasive breast carcinoma (pT1-2 pN0) were randomly assigned to 50 Gy/25 fr (5 weeks) or 30 or 28.5 Gy in 5 once-weekly fr of 6.0 or 5.7 Gy. The primary end point was change in photographic breast appearance at 2 and 5 years; secondary end points were physician assessments of NTE and local tumor control. Odds ratios (ORs) from longitudinal analyses compared regimens.

**RESULTS:**

A total of 915 women were recruited from 18 UK centers (2004-2007). Five-year photographs were available for 615/862 (71%) eligible patients. ORs for change in photographic breast appearance were 1.64 (95% CI, 1.08 to 2.49; *P* = .019) for 30 Gy and 1.10 (95% CI, 0.70 to 1.71; *P* = .686) for 28.5 Gy versus 50 Gy. α/β estimate for photographic end point was 2.7 Gy (95% CI, 1.5 to 3.9 Gy), giving a 5-fr schedule of 28 Gy (95% CI, 26 to 30 Gy) estimated to be isoeffective with 50 Gy/25 fr. ORs for any moderate/marked physician-assessed breast NTE (shrinkage, induration, telangiectasia, edema) were 2.12 (95% CI, 1.55 to 2.89; *P* < .001) for 30 Gy and 1.22 (95% CI, 0.87 to 1.72; *P* = .248) for 28.5 Gy versus 50 Gy. With 9.9 years median follow-up, 11 ipsilateral breast cancer events (50 Gy: 3; 30 Gy: 4; 28.5 Gy: 4) and 96 deaths (50 Gy: 30; 30 Gy: 33; 28.5 Gy: 33) have occurred.

**CONCLUSION:**

At 10 years, there was no significant difference in NTE rates after 28.5 Gy/5 fr compared with 50 Gy/25 fr, but NTE were higher after 30 Gy/5 fr. Results confirm the published 3-year findings that a once-weekly 5-fr schedule of whole-breast radiotherapy can be identified that appears to be radiobiologically comparable for NTE to a conventionally fractionated regimen.

## INTRODUCTION

Ten-year results of 4 randomized trials totaling > 7,000 patients confirm the safety and efficacy of hypofractionated radiotherapy after primary surgery for early breast cancer.^1-4^ The UK START-B and Ontario trials established 15- and 16-fraction schedules as new standards of care delivered over 21-22 days.^5-7^ Sensitivity to fraction size was tested in the START pilot and START-A trials by controlling for treatment time, generating an α/β estimate of 3.5 Gy (95% CI, 1.2 to 5.7) for tumor control, comparable to that for late adverse effects.^2,4,8^ Fifteen- or 16-fraction regimens are unlikely to represent the clinical limits of hypofractionation, and 3-year adverse effects of 5-fraction schedules in the UK FAST trial were reported in 2011.^9^ In FAST, 5.7 or 6.0 Gy once weekly were tested against 50 Gy in 25 fractions, the standard of care at the time. The explanatory trial design allowed interpolation between 2 5-fraction schedules that suggested a schedule equivalent to 50 Gy in 25 fractions in terms of late adverse effects. Five fractions of 5.7 and 6.0 Gy were predicted to be radiobiologically equivalent to 25 fractions of 2.0 Gy, assuming α/β values of 3.0 and 4.0 Gy for late normal tissue responses and tumor control, respectively.^10^ At a median follow-up of 3 years, 28.5 Gy in 5 fractions was comparable to 50 Gy in 25 fractions and milder than 30 Gy in 5 fractions in terms of adverse effects in the breast.^9^ This manuscript presents the 5-year results for change in photographic breast appearance and physician assessments of breast normal tissue effects (NTE) up to 10 years after radiotherapy, as well as breast cancer disease events.

**CONTEXT**

**Key Objective**
To test the reduction in total dose of adjuvant whole-breast radiotherapy delivered by 5 once-weekly fractions needed to match the late adverse effects of a standard 25-fraction schedule.
**Knowledge Generated**
A once-weekly 5-fraction schedule of 28 Gy is estimated to be radiobiologically equivalent to 50 Gy in 25 fractions in terms of late adverse effects at 10 years of follow-up.
**Relevance**
α/β estimates for late adverse effects are consistent with historical estimates of fraction size sensitivity in patients prescribed adjuvant whole-breast radiotherapy and can be used to inform additional trials of accelerated hypofractionation. Five-year results from the UK FAST-Forward trial have confirmed the efficacy of a 5-fraction schedule delivered in 1 week in terms of local tumor control. Our findings from the FAST trial may be relevant to the needs of patients who are unable to comply with or gain access to standard 25-, 16-, 15- or 5-fraction schedules, and in whom once-weekly treatment is preferable.

## METHODS

### Patients

FAST is a multicenter, phase III randomized controlled trial. Full details of trial design, eligibility criteria, radiotherapy planning and delivery, and study procedures have been presented previously (Protocol, online only).^9^ Eligible patients were women having invasive early breast cancer age ≥ 50 years, pathologic tumor size < 3 cm, axillary node negative, breast-conserving surgery with complete microscopic resection, and whole-breast radiotherapy. Patients requiring mastectomy, lymphatic radiotherapy, tumor bed boost, or cytotoxic therapy were ineligible.

Patients were randomly assigned (1:1:1) to receive 50 Gy in 25 fractions of 2.0 Gy, 30 Gy in 5 once-weekly fractions of 6.0 Gy, or 28.5 Gy in 5 once-weekly fractions of 5.7 Gy. Random assignment was performed by telephone or fax from the recruiting center to the Clinical Trials and Statistics Unit, Institute of Cancer Research, London. Computer-generated random permuted blocks stratified by participating center were used. Treatment allocation was not blinded because of the nature of the intervention.

All patients provided written informed consent. FAST (CRUKE/04/015) was approved by the national South-West Multicentre Research Ethics Committee (04/MRE06/17) and the local ethics committees of participating centers. FAST was sponsored by The Institute of Cancer Research and is registered as an International Standard Randomized Controlled Trial (ISRCTN62488883).

### Radiotherapy

Patients lay supine on an inclined plane in a position that remained unchanged during imaging/simulation and treatment, verified by orthogonal laser beams. Clinical target volume included soft tissues of the whole breast down to deep fascia but not including underlying muscle, ribcage, overlying skin, or excision scar. Planning target volume included the entire breast with 1-cm margins to palpable breast tissue. Medial and lateral borders did not normally extend beyond the anterior midline or the midaxilla. Margins were reduced in selected patients if the tumor bed did not encroach, to exclude or reduce the volume of heart and/or lung within the high-dose volume. The deep margin extended down to the deep fascia.

Transverse cross-sections of the patient were taken through the center of the planning target volume; a minimum of 5 slices was recommended, spaced appropriately. Sixteen out of 18 centers used full-dose compensation with computerized tomography; others used optical outlining devices capturing the central external contour supplemented by 2 additional outlines collected 1 cm inside the superior field border and 1 cm superior to the inframammary fold.^11^ The maximum thickness of lung included in the tangential field was 2 cm; cardiac shielding used multileaf collimator (MLC) or other technique. The dose distribution across the target volume was modified to ensure homogeneity within ICRU50/62 guidelines.^12^ Doses were prescribed to the reference point at/near the center of the target volume. Maximum and minimum doses were ≤ 10% of doses on the central plane after full dose compensation; where full dose compensation was not possible, maximum doses in the superior plane and plane through the inframammary fold were recorded. Three main dose compensation methods were used to improve dose homogeneity: (1) physical breast compensators, (2) simple forward-planned intensity-modulated radiation therapy (IMRT) MLC segment fields/field-in-field technique, and (3) inverse-planned IMRT MLC segment fields.^13^

### Outcome Assessment

The primary end point was change in photographic breast appearance. Secondary end points were physician assessments of radiation-induced breast changes and ipsilateral disease in the breast (relapse or new primary).

**FIG A1.**
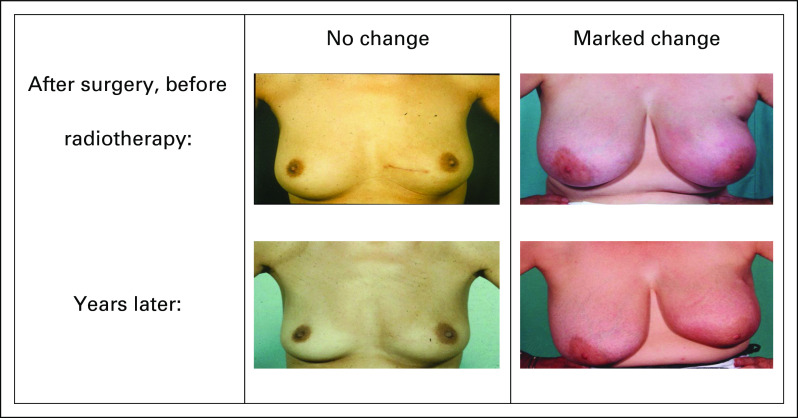
Examples of no change and marked change in photographic breast appearance.

Photographs were taken at baseline and 2 and 5 years after radiotherapy. Change in photographic breast appearance compared with the postsurgical (preradiotherapy) baseline was scored on a qualitative 3-point scale (no, mild, or marked change), on the basis of changes in size, shrinkage, and shape. Patients were ineligible for additional photographic assessments after breast reconstructive surgery and after additional ipsilateral disease. All photographs were scored by at least 2 observers blind to patient identity and treatment allocation following procedures established in the START Trials^14^ (Appendix Figure A1, online only). Because a number of years had elapsed since the scoring of the 2-year photographs for the previous publication,^9^ these were rescored along with the 5-year photographs to ensure consistency of assessment criteria (Appendix Table A1, online only). Breast size and surgical deficit were assessed from the baseline photographs using a qualitative 3-point scale (small, medium, large), with surgical deficit expressed relative to the contralateral breast size.

Late-onset NTE in the breast (shrinkage, induration, telangiectasia, edema) were assessed by physicians at annual follow-up and graded on a 4-point scale for the treated breast relative to the contralateral breast (none, a little, quite a bit, or very much; interpreted as none, mild, moderate or marked). Incidence of symptomatic rib fracture, symptomatic lung fibrosis, and ischemic heart disease was recorded. Physicians were not blinded to randomized treatment allocation. No patient-reported outcomes were assessed within the FAST trial. Clinical assessments of acute skin toxicity have been previously reported.^9^

Ipsilateral disease was defined as a malignancy (invasive or ductal carcinoma in situ) presenting anywhere in the ipsilateral breast parenchyma and/or overlying skin, whether considered ipsilateral breast relapse or new primary tumor. Data on first regional relapse (axilla, supraclavicular fossa, and internal mammary chain), distant metastases, new primary cancer, and death were also collected.

### Statistical Considerations

Using START pilot trial results,^2^ an average 2-year rate of mild or marked change in photographic breast appearance for the test groups of 20% was assumed, allowing a sample size of 900 to detect a 10% difference in the prevalence of change in photographic breast appearance between test dose levels with 90% power, 2-sided α = 0.05, allowing for 10% loss to follow-up/unevaluable. The trial was not statistically powered to test for differences in local tumor control.

Scores for change in photographic breast appearance at 2 and 5 years were modeled using generalized estimating equations (GEE).^15^ Mild and marked categories were combined, because marked change was rare. Pairwise comparisons of mild/marked change between schedules were described by odds ratios (ORs, with 95% CI) obtained from the GEE models and the Wald test.

Cross-sectional analyses of physician-assessed breast NTE at 5 and 10 years compared frequencies of moderate/marked effects versus none/mild between pairs of schedules using risk ratios and risk differences (with 95% CI), and Fisher’s exact test. Longitudinal analyses of moderate/marked physician-assessed NTE (*v* none/mild) used GEE models including all annual assessments, comparing schedules across the whole follow-up period using OR (with 95% CI) and the Wald test; a term representing years of follow-up was included, enabling time trends to be modeled. Survival analysis methods analyzed time to first moderate/marked physician-assessed NTE, including Kaplan-Meier plots and estimates of cumulative incidence rates. Hazard ratios (HRs, with 95% CI) were obtained from Cox proportional hazards regression, and schedules were compared using the log-rank test. Inconsistencies between the GEE and Cox models for some end points appeared to be due to more patients in the 28.5-Gy group having only 1 event, which has a greater influence on the time-to-event analysis (where only 1 event is needed) compared with the longitudinal models including all events over follow-up.

Kaplan-Meier estimates (with 95% CI) of 5- and 10-year cumulative incidence of ipsilateral disease in the breast were calculated, and HR (with 95% CI) compared schedules obtained from Cox proportional hazards regression, with patients censored at date of distant metastases, new primary cancer (contralateral breast or nonbreast), death, or date of last follow-up.

Estimates of the α/β ratio for late NTE were obtained by fitting GEE models to all follow-up assessments (photographic and physician), including terms for total dose and total dose multiplied by fraction size. The α/β ratio was calculated as estimate for total dose/estimate for total dose × fraction size, with 95% CI estimated from the model (lower confidence limits were truncated at zero when the calculated limit was negative). Isoeffect doses in 2.0-Gy equivalents were calculated for the experimental schedules, and the 5-fraction schedule estimated to be isoeffective with 50 Gy/25 fractions was derived.

All analyses were performed on an intention-to-treat basis, from a database snapshot taken on July 17, 2018; Stata version 15 (StataCorp, College Station, TX) was used.

## RESULTS

**FIG A2.**
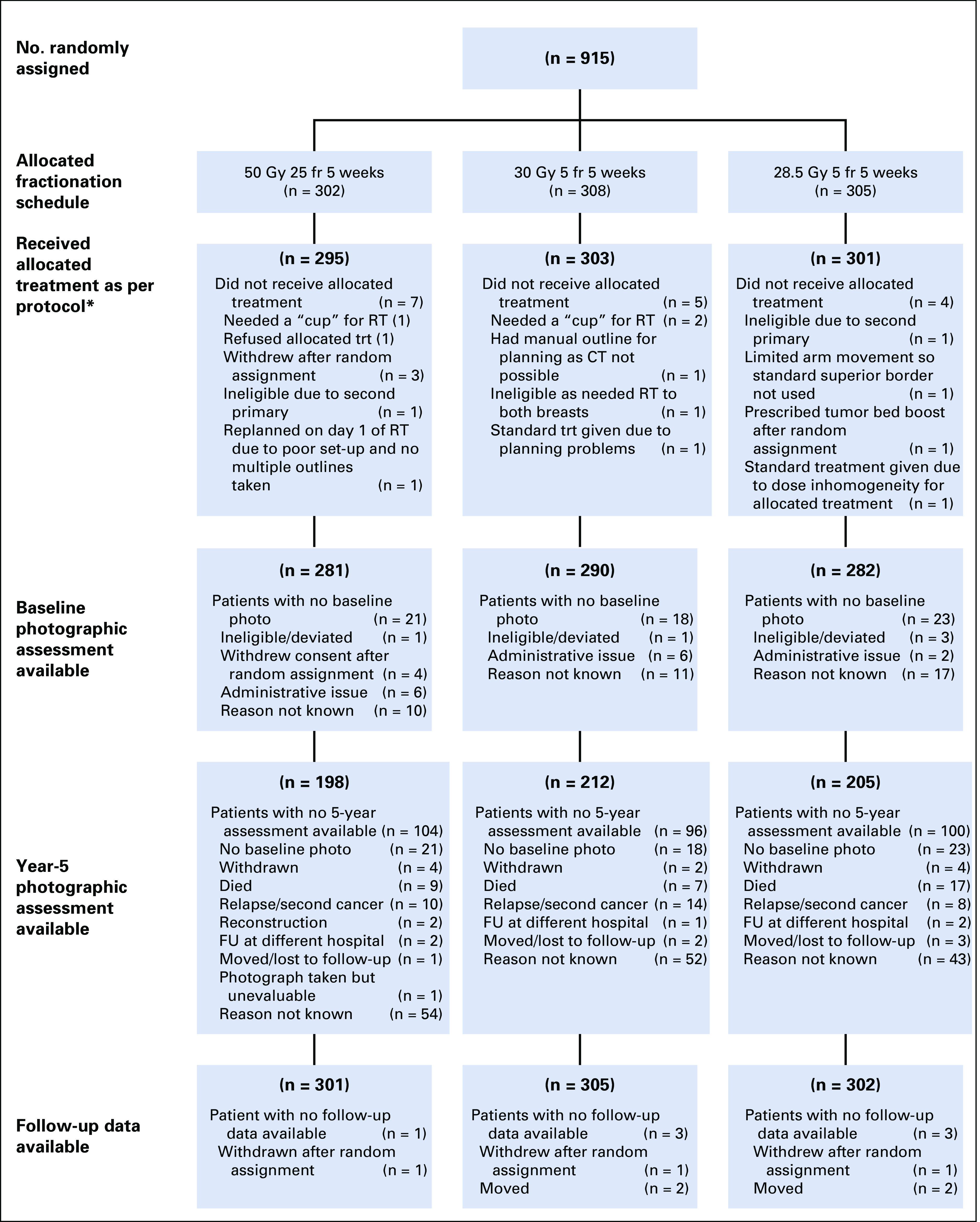
FAST Trial profile. FU, follow-up; fr, fraction; RT, radiotherapy; trt, treatment. (*) Only major treatment deviations are listed. Minor deviations due to public holidays, machine service days, and machine breakdowns not included.

A total of 915 women were recruited from October 2004 to March 2007 from 18 UK radiotherapy centers. Baseline clinical and demographic details were reported previously^9^ (Appendix Table A2>, online only). Mean age at random assignment was 62.9 years (range, 50-88 years), mean pathologic tumor size was 1.3 cm (range, 0.1-3.0 cm), 34% of patients had a grade 1 tumor, and 88.4% of patients were scheduled to receive adjuvant endocrine therapy. At the time of analysis, median follow-up was 9.9 years (interquartile range, 8.3-10.1 years). Of patients alive and disease free, assessments of change in photographic breast appearance were available for 732/901 (81%) patients at 2 years and 615/862 (71%) at 5 years (Appendix Figures A1 and A2).

At 5 years, 489/615 (79.5%) patients had no change in photographic breast appearance, 109 (17.7%) had mild change, and 17 (2.8%) had marked change. Rates of mild/marked change in photographic breast appearance at 2 or 5 years were statistically significantly higher for 30 Gy compared with 50 Gy (OR, 1.64; 95% CI, 1.08 to 2.49; *P* = .019) but not significantly different for 28.5 Gy and 50 Gy (OR, 1.10; 95% CI, 0.70 to 1.71; *P* = .686; Table 1). Rates of mild/marked change in photographic breast appearance were slightly higher for 30 Gy compared with 28.5 Gy (*P* = .052).

**TABLE 1.**
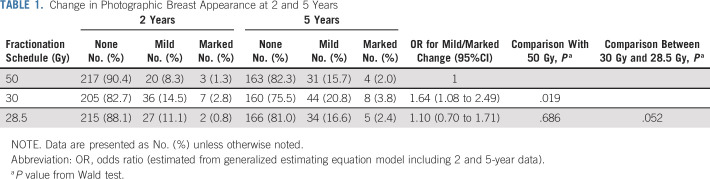
Change in Photographic Breast Appearance at 2 and 5 Years

**FIG 1.**
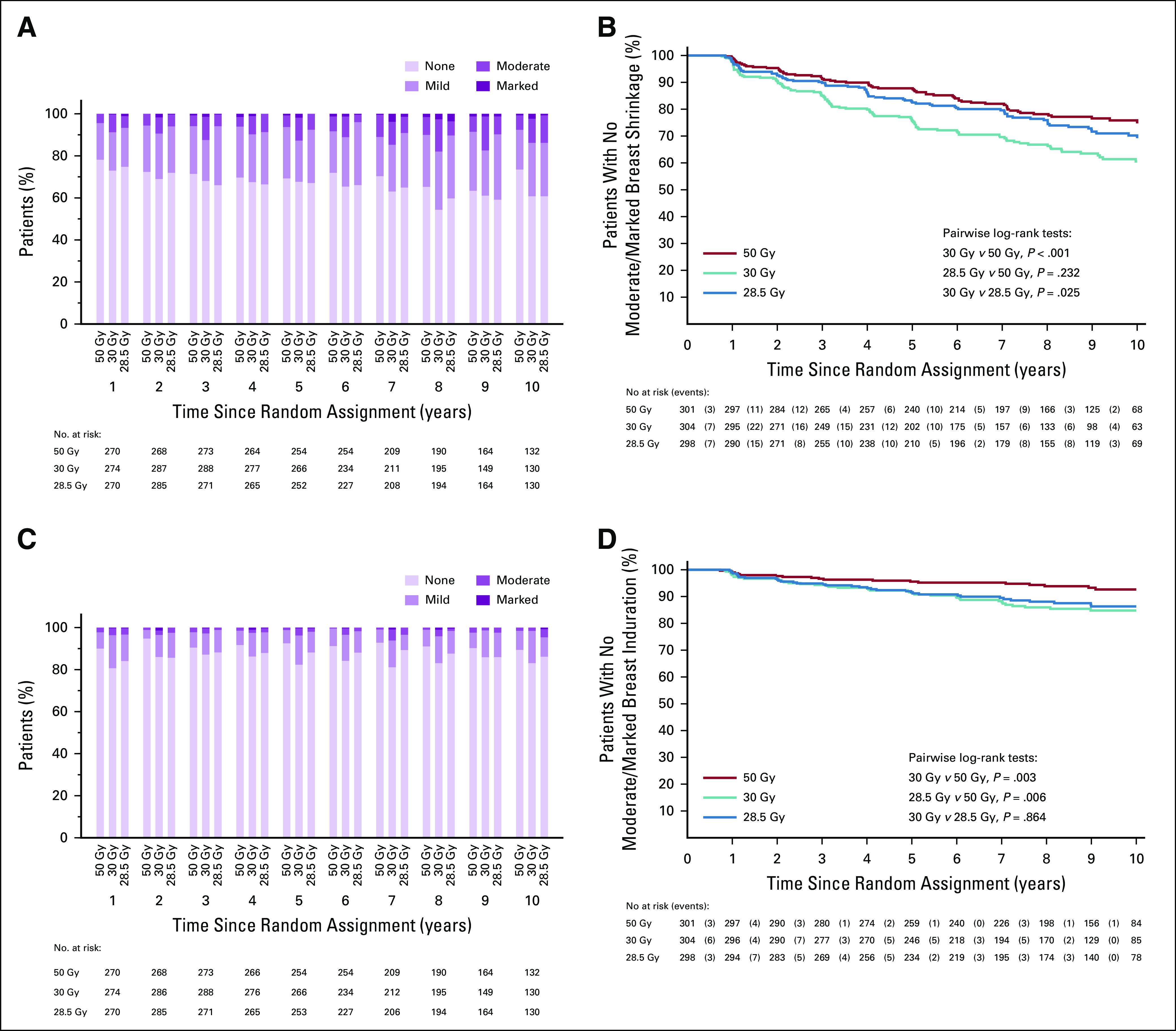

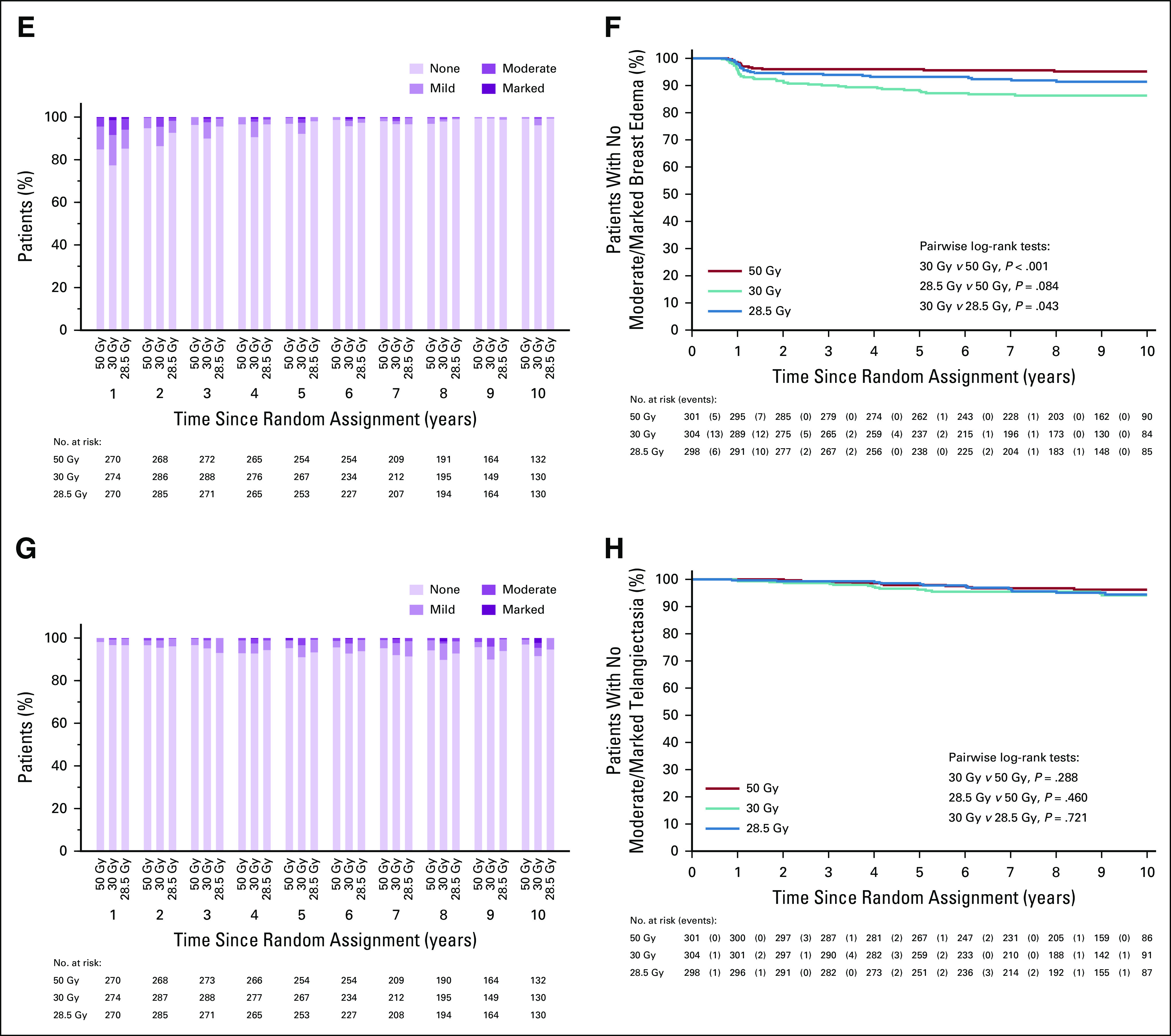
Physician assessments of late normal tissue effects. (A) Breast shrinkage to 10 years; (B) time to first reported moderate/marked breast shrinkage; (C) breast induration to 10 years; (D) time to first reported moderate/marked breast induration; (E) breast edema to 10 years; (F) time to first reported moderate/marked breast edema; (G) telangiectasia to 10 years; and (H) time to first reported moderate/marked telangiectasia.

Any moderate/marked physician-assessed NTE in the breast (shrinkage, induration, telangiectasia, edema) was reported for 92/774 (11.9%) at 5 years and 55/392 (14.0%) at 10 years (Table 2). The most prevalent individual effect was breast shrinkage (Fig 1). Five-year prevalence of any moderate/marked breast NTE was estimated to be 10% higher (95% CI, 5% to 16%) for 30 Gy versus 50 Gy (*P* < .001), with no statistically significant difference between 28.5 Gy and 50 Gy (2%; 95% CI, −2% to +7%; *P* = .349). At 5 years, risk ratios for moderate/marked breast shrinkage versus 50 Gy were 2.03 (95% CI, 1.15 to 3.58; *P* = .017) for 30 Gy and 1.20 (95% CI, 0.63 to 2.27; *P* = .604) for 28.5 Gy. There were no statistically significant differences between schedules in 5-year prevalence of moderate/marked breast induration, telangiectasia, and breast edema, nor in 10-year prevalence of any moderate/marked effects, with few marked events (Table 2). At 10 years, the estimated absolute differences in prevalence of any moderate/marked breast NTE compared with 50 Gy were 9% (95% CI, 1% to 18%: *P* = .032) for 30 Gy and 5% (95% CI, −2% to +13%; *P* = .184) for 28.5 Gy.

**TABLE 2.**
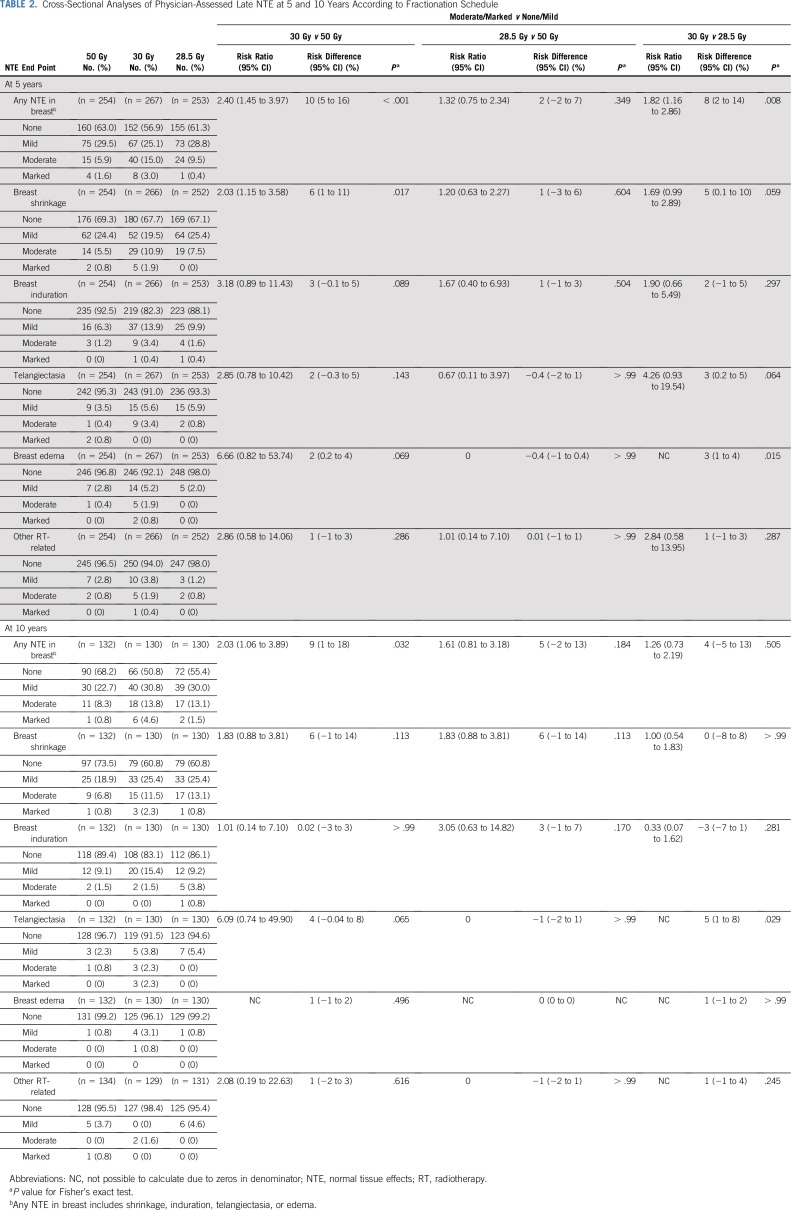
Cross-Sectional Analyses of Physician-Assessed Late NTE at 5 and 10 Years According to Fractionation Schedule

Five- and 10-year cumulative incidence rates of moderate/marked NTE in the breast were higher for 30 Gy compared with 50 Gy, with statistically significant differences for any NTE in the breast, breast shrinkage, breast induration, and breast edema (Fig 1; Appendix Table A3, online only). Cumulative incidence rates of any moderate/marked NTE in the breast and breast induration were significantly higher for 28.5 Gy versus 50 Gy.

Modeling all annual physician assessments over follow-up, rates of moderate/marked effects were statistically significantly higher for 30 Gy compared with 50 Gy (OR for any breast NTE, 2.12; 95% CI, 1.55 to 2.89; *P* < .001), but with no significant difference between 28.5 Gy and 50 Gy (OR, 1.22; 95% CI, 0.87 to 1.72; *P* = .248; Table 3). Statistically significant differences between the test schedules were found for breast shrinkage, telangiectasia, and breast edema, with higher rates for 30 Gy compared with 28.5 Gy. The prevalence of breast shrinkage and telangiectasia increased over time, with a decline in breast edema (Fig 1).

**TABLE 3.**
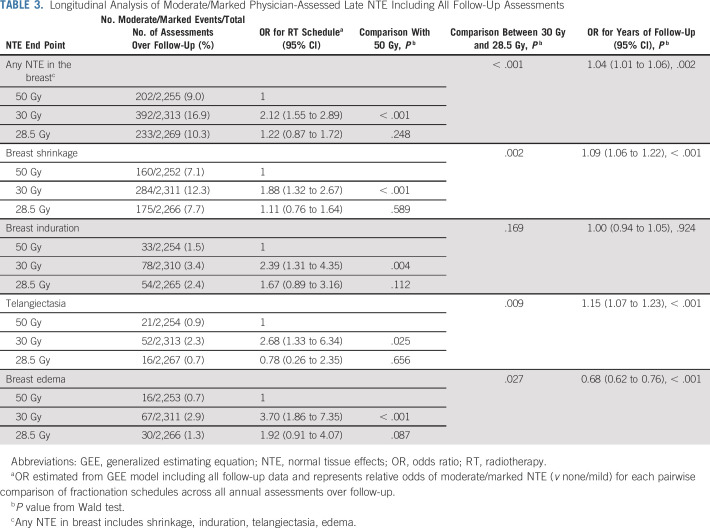
Longitudinal Analysis of Moderate/Marked Physician-Assessed Late NTE Including All Follow-Up Assessments

Change in photographic breast appearance gave an unadjusted α/β estimate of 2.7 Gy (95% CI, 1.5 to 3.9 Gy); adjusting for breast size and surgical deficit made little difference (Table 4). Using an α/β of 2.7 Gy, the isoeffect doses expressed in 2.0-Gy equivalents for 30 and 28.5 Gy in 5 fractions were approximately 56 and 51 Gy, respectively, and the once-weekly 5-fraction schedule estimated to be isoeffective with 50 Gy/25 fractions was 28 Gy (95% CI, 26 to 30 Gy). Estimates of α/β for physician-assessed NTE were consistent with the photographic end point (Table 4).

**TABLE 4.**
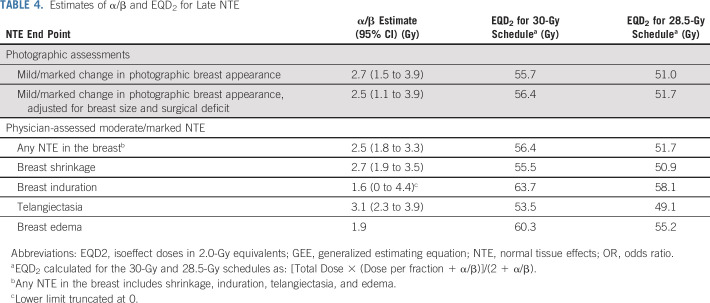
Estimates of α/β and EQD_2_ for Late NTE

A total of 123 patients (13.4%) were referred to a specialist for radiotherapy-related adverse effects, most frequently lymphedema, with similar rates between the schedules (Appendix Table A4, online only). Symptomatic rib fracture was reported for 11 patients (1.2%), symptomatic lung fibrosis for 8 (0.9%), and ischemic heart disease for 17 (1.9%), including 7 cases in patients treated for left-sided breast cancer (Appendix Table A5, online only).

Ipsilateral breast events were reported for 11/915 (1.2%) patients (50 Gy: 3; 30 Gy: 4; 28.5 Gy: 4), with estimated cumulative incidence rates of 0.7% (95% CI, 0.3% to 1.6%) at 5 years and 1.3% (95% CI, 0.7% to 2.3%) at 10 years (Table 5). A total of 96 patients (10.5%) have died (50 Gy: 30; 30 Gy: 33; 28.5 Gy: 33), including 25 (2.7%) breast cancer deaths (50 Gy: 7; 30 Gy: 8; 28.5 Gy: 10). Schedules appeared similar regarding breast cancer–related events, new primary cancers, or deaths, although numbers were small (Appendix Table A6, online only).

**TABLE 5.**
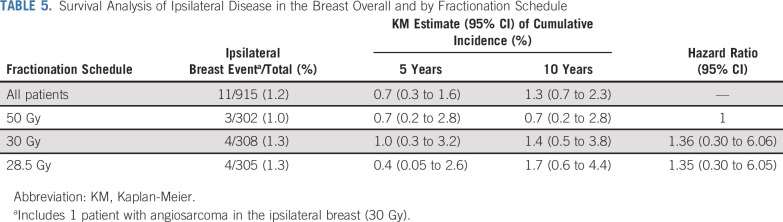
Survival Analysis of Ipsilateral Disease in the Breast Overall and by Fractionation Schedule

## DISCUSSION

The FAST Trial tested once-weekly 5-fraction schedules of whole-breast radiotherapy in terms of late NTE against a standard regimen of 50 Gy in 25 fractions. Patient eligibility focused on factors associated with a low absolute risk of local tumor relapse, as experienced by an older patient age group with early-stage pathologically node-negative disease.

Change in photographic breast appearance was the primary end point of late NTE as in the START trials, because breast appearance after breast cancer treatment is of importance to women, and photographs allow external assessors to control for baseline surgical deficit and to score postradiotherapy changes blind to treatment allocation.^14^ Marked change in photographic breast appearance in the FAST trial was rare. The low rates of change recorded in the FAST trial after 50 Gy incorporate the benefits of 3-dimensional dosimetry compared with 2-dimensional dosimetry used in the START and Ontario trials, as well as fewer women with large breast size included in FAST. Physician assessments, although not blinded to allocated treatment and hence potentially subject to bias, nevertheless provide a valuable assessment of late NTE from a different perspective to the photographs, and both sets of results contribute to the overall evidence from the trial. Annual physician assessments identified few moderate or marked effects over 10 years. The prevalence of breast shrinkage and telangiectasia increased over follow-up in FAST, as shown in other studies,^4,16^ whereas breast edema declined, consistent with patient-reported outcomes of the IMPORT LOW trial of partial breast radiotherapy.^17^ Incident cases of ischemic heart disease were rare, but longer follow-up is required to adequately monitor cardiac risk after breast radiotherapy.

The α/β estimates from FAST are consistent with the 10-year analysis of the START-A trial, which generated estimates around 3-4 Gy for late NTE in the breast.^4^ This consistency supports the validity of the linear-quadratic model for fraction sizes as high as 5.0-6.0 Gy. However, fractionation sensitivity might be slightly higher (α/β value slightly lower) than predicted by the model because of much lower rates of moist desquamation and later consequential late skin damage when larger fractions are used. Rates of patchy/confluent moist desquamation in the FAST trial after 50.0 Gy, 30.0 Gy, and 28.5 Gy were 11.7%, 2.7%, and 2.8%, respectively (including only 1 confluent case), confirming the well-established insensitivity of early-reacting self-renewal tissues to fraction size and the importance of total dose.^9,18^

The FAST trial was not powered for formal statistical comparison of local tumor control; the 10-year cumulative incidence estimate was 1.3%, in keeping with the low-risk population for which the trial was designed. The extremely low number of local tumor events reflects the patient demographics, tumor characteristics, careful attention to microscopic excision margins, the use of adjuvant endocrine therapy, and high-quality radiotherapy. Deaths from other causes were the most frequent consequential event.

The FAST trial was conceived in the early 2000s, and since then the UK^5^ and international standard^7^ has become 40 Gy in 15 fractions over 3 weeks or similarly hypofractionated. On the basis of an α/β value of 2.7 Gy, the 15-fraction regimen is equivalent to 45.7 Gy in 2.0-Gy equivalents. In response to 10-year results of the START and Ontario trials, 15- or 16-fraction regimens are the preferred dose-fractionation options for whole-breast radiotherapy according to the American Society of Radiation Oncology.^7^ FAST informed the design of the UK phase III FAST-Forward trial testing 2 dose levels of a 5-fraction schedule delivered in 1 week compared with 40 Gy in 15 fractions in women prescribed adjuvant radiotherapy to whole breast or postmastectomy chest wall after primary surgery for early breast cancer. FAST-Forward demonstrated non-inferiority of the 5-fraction schedules in terms of 5-year ipsilateral tumor control, with similar rates of late NTE up to 5 years for the 26 Gy 5-fraction schedule compared with 40 Gy in 15 fractions, and is already considered standard in many UK radiotherapy departments.^19^ A substudy within FAST-Forward tests the same dose schedules as the main trial in patients who also require radiotherapy to the axilla and/or supraclavicular fossa.

In conclusion, the FAST trial identifies a 5-fraction schedule estimated to be radiobiologically equivalent to the 25-fraction standard in terms of late NTE. Identification of a 5-fraction schedule equivalent with respect to tumor control is being evaluated in the UK FAST-Forward trial. Although not powered for tumor control, the FAST trial suggests that for patients at low risk of relapse and for whom daily visits over 3 or 5 weeks are not possible because of frailty or comorbidities, 28 Gy in 5 fractions as a once-weekly schedule might be an appropriate alternative to no treatment.

## Figures and Tables

**TABLE A1.**
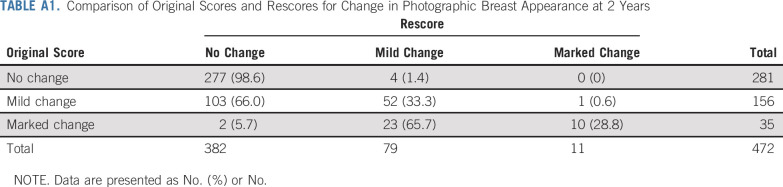
Comparison of Original Scores and Rescores for Change in Photographic Breast Appearance at 2 Years

**TABLE A2.**
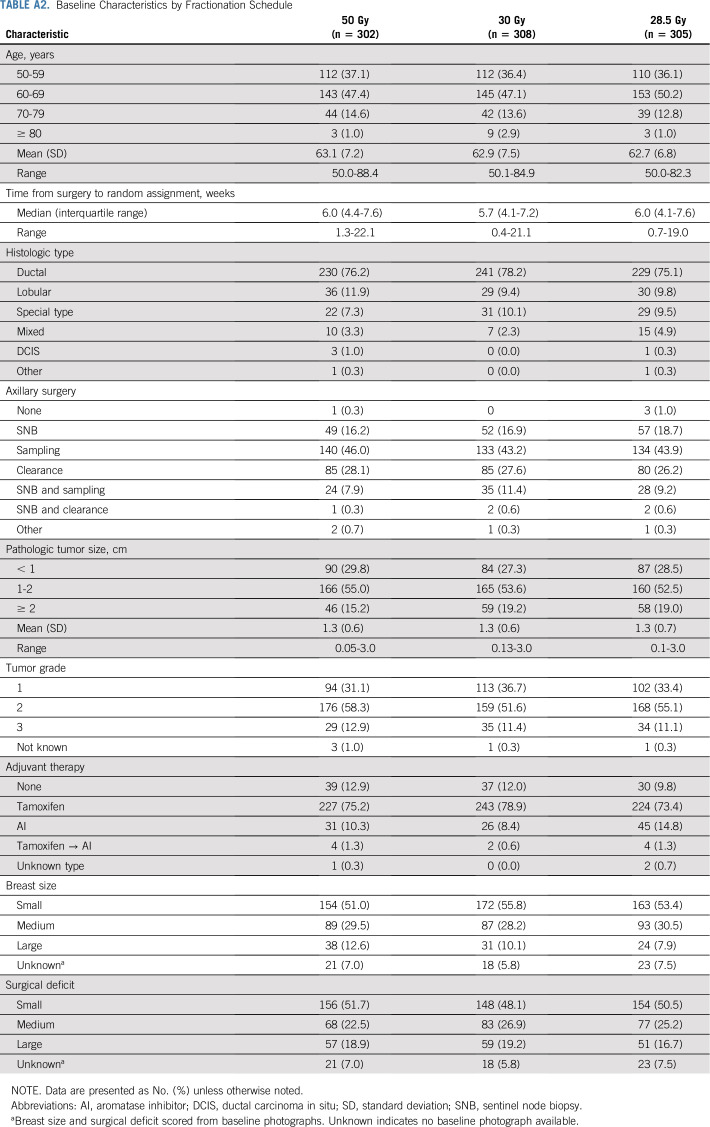
Baseline Characteristics by Fractionation Schedule

**TABLE A3.**
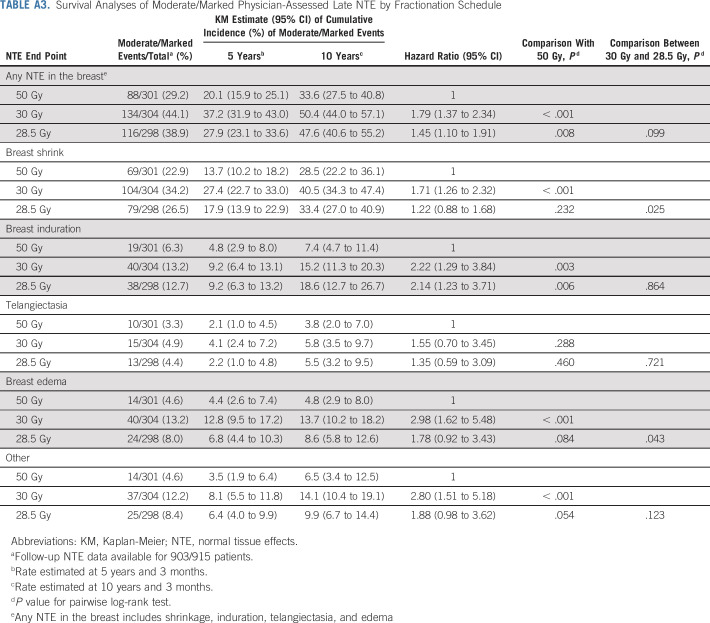
Survival Analyses of Moderate/Marked Physician-Assessed Late NTE by Fractionation Schedule

**TABLE A4.**
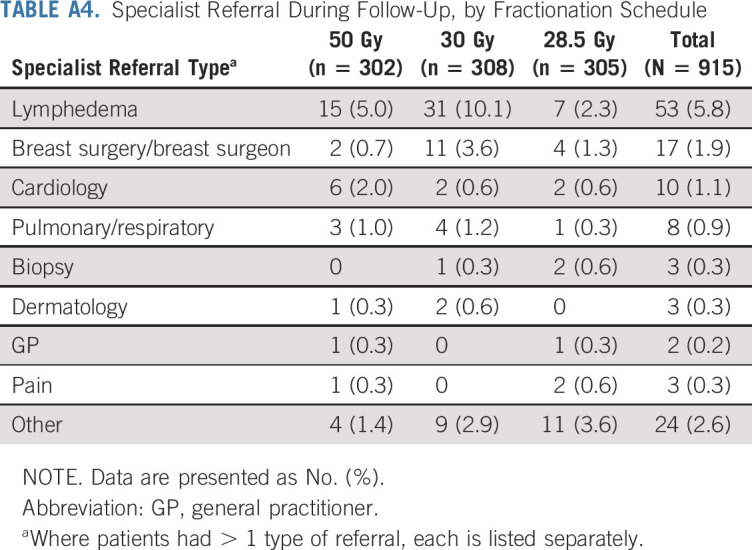
Specialist Referral During Follow-Up, by Fractionation Schedule

**TABLE A5.**
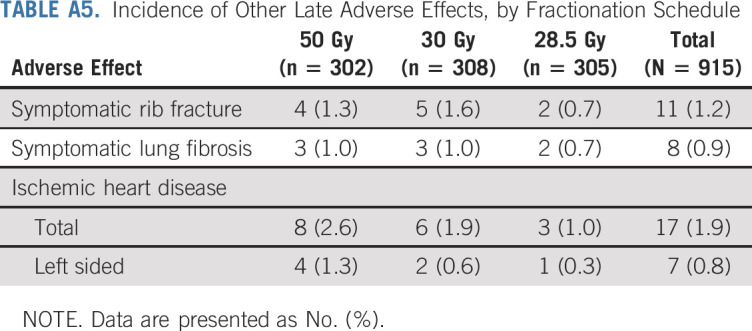
Incidence of Other Late Adverse Effects, by Fractionation Schedule

**TABLE A6.**
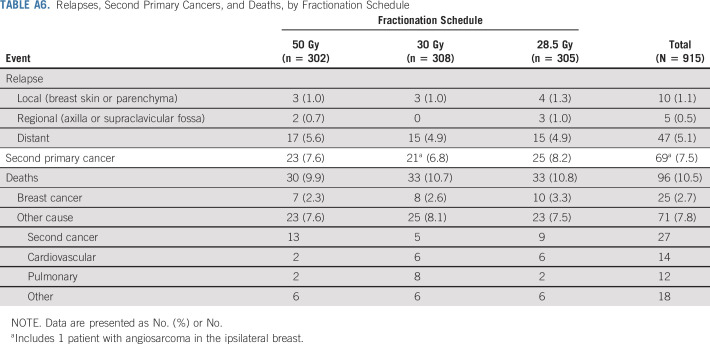
Relapses, Second Primary Cancers, and Deaths, by Fractionation Schedule

## Appendix

### Principal and Main Co-Investigators According to Center (No. of patients recruited)

Cheltenham General Hospital, Cheltenham (n = 2), K. Benstead, J. R. Owen; Gloucester Royal Hospital, Gloucester (n= 2), K. Benstead; Worcestershire Royal Infirmary, Worcester (n = 6), J. Bowen, R. Counsell; Christie Hospital, Manchester (n = 12), A. Stewart; Clatterbridge Centre for Oncology, Bebington (n = 6), I. Syndikus; Warrington and Halton Hospitals, Warrington (n = 18), I. Syndikus; Ipswich Hospital, Ipswich (n = 17), E. Sherwin; Leeds General Hospital, Leeds (n = 5), S. Kumar; Mid-Yorks Hospitals, Wakefield (n = 4), S. Kumar, F. Roberts; Norfolk and Norwich University Hospital, Norwich (n = 27), A. Harnett, A. Bulman; James Paget, Norfolk and Norwich (n = 25), A. Harnett, A. Bulman; University Hospital of North Staffordshire, Stoke-on-Trent (n = 112), A. M. Brunt, A. Al Niaimi; Royal Marsden Hospital, Sutton (n = 75), J. R. Yarnold, D. Tait, A. Rostom, M. Dryzmala; Royal Cornwall Hospital, Truro (n = 109), D. Wheatley, A. Thomson, T. Hurst; Royal Devon and Exeter Hospital, Exeter (n = 61), A. Goodman, A. Hong, P. Bliss; North Devon Hospital, (n = 20), A. Hong; Burnley General Hospital, Burnley (n = 14), M. Hogg, W. Appel; Blackpool Royal Infirmary, Blackpool (n = 6), Royal Preston, A. Hindley, S. Susnerwala; Royal Shrewsbury Hospital, Shrewsbury (n = 36), R. K. Agrawal; Southend General Hospital, Southend (n = 66), A. Robinson; Basildon University Hospital, Basildon (n = 3), C. Trask; Torbay District General Hospital, Torbay (n = 58), P. Bliss, A. Goodman; Velindre Hospital, Cardiff (n = 42), J. Abraham, C. Gaffney, P. J. Barrett-Lee; Royal Gwent Hospital (n = 7), J. Abraham, C. Gaffney, P. J. Barrett-Lee; Royal Glamorgan Hospital, (n = 4), J. Abraham; Royal Sussex County Hospital, Brighton (n = 34), D. Bloomfield, R. Simcock; Worthing Hospital, Worthing (n = 41), S. Mitra; Eastbourne Hospital, Eastbourne (n = 2), A. Robinson; Queens Hospital, Romford (n = 9), M. Quigley, E. Sims; Beatson Oncology Centre, Glasgow (n = 85), A. Alhasso, D. Ritchie; Victoria Infirmary, Glasgow (n = 2), A. Alhasso; Crosshouse Hospital, Kilmarnock (n = 5), A. Alhasso, D. Ritchie.

### Methods

Photographs were scored by a team comprising 2-3 observers (2 consultant clinical oncologists including J.R.Y., Chief Investigator of FAST Trial, and a research manager in the chief investigator’s research team). Each scoring session began with a review of photographs previously scored, followed by scoring of the new photographs; sessions generally lasted for 1 day. As previously described,^12^ observers conferred and agreed on a score by consensus. The same processes were followed for the 5-year photographs as for the original 2-year photograph scoring, with one change of personnel (clinical oncologist).

The categories of mild and marked change were assessed qualitatively, as it was not possible to quantitatively measure breast shrinkage from the photographs. Examples of no change and marked change in photographic breast appearance are shown in Appendix Figure A1.

### Update of 2-Year Results

Three additional 2-year photographs were scored since the 2011 publication,^9^ taking the total at year 2 to 732.

When the year 5 photographs were scored, it was noted that the overall prevalence of mild and marked changes was unexpectedly lower than reported at 2 years in the 2011 publication. Marked changes in particular would not be expected to reverse, except for some patients with marked breast edema. Because there was no objective measure used, such as a quantitative measurement of breast shrinkage, for example, it is considered more likely that perceptions of radiotherapy-related changes changed over the long time period since the 2-year photographs were originally scored, causing discrepancies between the published 2-year results and the 5-year results reported here. Hence it was decided to rescore all 2-year photographs originally scored as mild or marked change, together with a random sample of those originally scored as no change.

Overall, 472 paired scores (original and rescore) were available for year 2. The number of scores expected to agree by chance were calculated, along with the weighted κ statistic to test agreement between the pairs.

There were fewer mild and marked changes in the rescores at year 2 (Appendix Table A1). The number of pairs of scores expected to agree by chance were 227 (no change), 26 (mild), 0.8 (marked) (ie, observed agreement for mild and marked changes was higher than would be expected by chance; weighted κ, 0.46; “moderate” agreement; SE 0.03).

In summary, although the observed agreement between the original scores and the rescores for mild and marked changes was higher than would be expected by chance, it was decided to use the rescores for all analyses presented in this article, as the level of agreement overall was only moderate. The number of patients with mild/marked change in photographic breast appearance at 2 years reported here is less than in the 2011 article, but conclusions regarding differences between the fractionation schedules are as before.^9^
